# The emergent integrated network structure of scientific research

**DOI:** 10.1371/journal.pone.0216146

**Published:** 2019-04-30

**Authors:** Jordan D. Dworkin, Russell T. Shinohara, Danielle S. Bassett

**Affiliations:** 1 Department of Biostatistics, Epidemiology, and Informatics, Perelman School of Medicine, University of Pennsylvania, Philadelphia, PA, United States of America; 2 Department of Bioengineering, University of Pennsylvania, Philadelphia, PA, United States of America; 3 Department of Physics & Astronomy, University of Pennsylvania, Philadelphia, PA, United States of America; 4 Department of Electrical & Systems Engineering, University of Pennsylvania, Philadelphia, PA, United States of America; 5 Department of Neurology, University of Pennsylvania, Philadelphia, PA, United States of America; University of Bristol, UNITED KINGDOM

## Abstract

Scientific research is often thought of as being conducted by individuals and small teams striving for disciplinary advances. Yet as a whole, this endeavor more closely resembles a complex and integrated system of people, papers, and ideas. Studies of co-authorship and citation networks have revealed important structural properties of researchers and articles, but currently the structure of scientific ideas themselves is not well understood. In this study, we posit that topic networks may be a useful framework for revealing the nature of conceptual relationships. Using this framework, we map the landscape of interconnected research topics covered in the multidisciplinary journal *PNAS* since 2000, constructing networks in which nodes represent topics of study and edges give the extent to which topics occur in the same papers. The network displays small-world architecture, characterized by regions of dense local connectivity with sparse connectivity between them. In this network, dense local connectivity additionally gives rise to distinct clusters of related topics. Yet notably, these clusters tend not to align with assigned article classifications, and instead contain topics from various disciplines. Using a temporal graph, we find that small-worldness has increased over time, suggesting growing efficiency and integration of ideas. Finally, we define two measures of interdisciplinarity, one of which is found to be positively associated with *PNAS*’s impact factor. Broadly, this work suggests that complex and dynamic patterns of knowledge emerge from scientific research, and that structures reflecting intellectual integration may be beneficial for obtaining scientific insight.

## Introduction

The practice of scientific research represents the collective effort of humans to acquire information, generate insight, and disseminate knowledge. Although scientific inquiry has been carried out for centuries, the recent expansion of meta-data collection has allowed a robust body of literature to develop around the scientific study of science itself. This work has led to advances in predicting the success of scientific papers and authors [1, 2], found that articles often do not fit into existing disciplinary boundaries [3, 4], and provided empirical fuel for the debate over interdisciplinary research [5–8]. Yet much remains unknown about the nature of the large-scale scientific system that emerges from individuals’ intellectual and social incentives. It is especially unclear what features of this system may make it more or less effective at producing insights.

In recent years, network analysis has provided a particularly useful framework for beginning to reveal the structure and evolution of the emergent scientific landscape. The tools of this growing discipline have facilitated greater understanding of the roles of specific authors or papers in co-authorship and citation networks. Network measures can predict authors’ future collaboration patterns [9, 10], and can help identify turning points in the literature [11]. While the fine-scale topology of such networks differs by scientific discipline [12, 13], many network architectures display similar global properties. One such commonly shared property is small-world architecture [13, 14], which reflects high local clustering within specialty, potentially supporting development and refinement within sub-fields, combined with efficient paths that connect distant areas, providing outlets for innovation and information sharing.

Although co-authorship and citation networks have provided much insight into the properties of the scientific community, their dependence on authors’ social network structures makes them an indirect window into the structure of scientific knowledge. *Topic* networks, which reflect the relations between scientific ideas, offer an opportunity to fill this gap. Existing studies of topic networks have tended to focus on manual inspection of network appearance or node-level trends, with only occasional analyses of the networks’ large-scale features [15–21]. Yet, the operationalization of science as a set of interconnected ideas provides a unique opportunity to study how research topics are related within and across scientific disciplines, how these topics and their relationships grow and change over time, and how these changes may influence the extent to which scientists engage with the literature.

In this study, we seek to demonstrate the potential value of topic networks for understanding the large-scale network structure of scientific concepts. We present a generalizable methodological framework for studying topic relationships within scientific literature, and apply it to a network of topics covered in *PNAS* since the year 2000. In the presented analysis, network nodes reflect specific words or phrases, and network edges reflect the extent of co-occurrence within article abstracts and keyword sections. Using the resultant weighted, undirected network of scientific topics, we conduct an exploratory investigation of the static and dynamic natures of the topic network, focusing on four specific hypotheses.

First, building on findings from co-authorship and citation networks [13, 14], we hypothesize that the topic network will demonstrate non-random, small-world structure. Second, based on prior studies that performed latent topic modeling [3, 4], we hypothesize that the community structure of the network will deviate significantly from disciplinary classifications. Third, as collaboration has crossed national boundaries and broadly increased in recent years [22, 23], we hypothesize that over time the network will show greater bridging across topic communities. Finally, although the benefits of interdisciplinarity for individual papers are debated [5–8], we seek to investigate whether the topic network’s interdisciplinarity is associated with the overall amount of engagement the component literature receives, as measured by *PNAS*’s impact factor.

## Design and application

For this study, we used data from 65,290 articles published in *PNAS* between 2000 and 2017 to create a network of research topics. Though limited in scope, the choice to apply this framework to data from a single multidisciplinary journal was made for two critical reasons. First, the regularity with which disciplinary classifications are applied to articles sometimes varies across journals, and each journal has its own set of disciplinary classifications. The use of data from one journals facilitates a consistent and standardized system of classifications, allowing for investigation into the extent to which research topics do or do not cross disciplinary boundaries. Second, as the external relevance of the topic network was of interest, a single journal was desirable in order to draw connections between network structure and journal impact factor over time.

**Fig 1:**
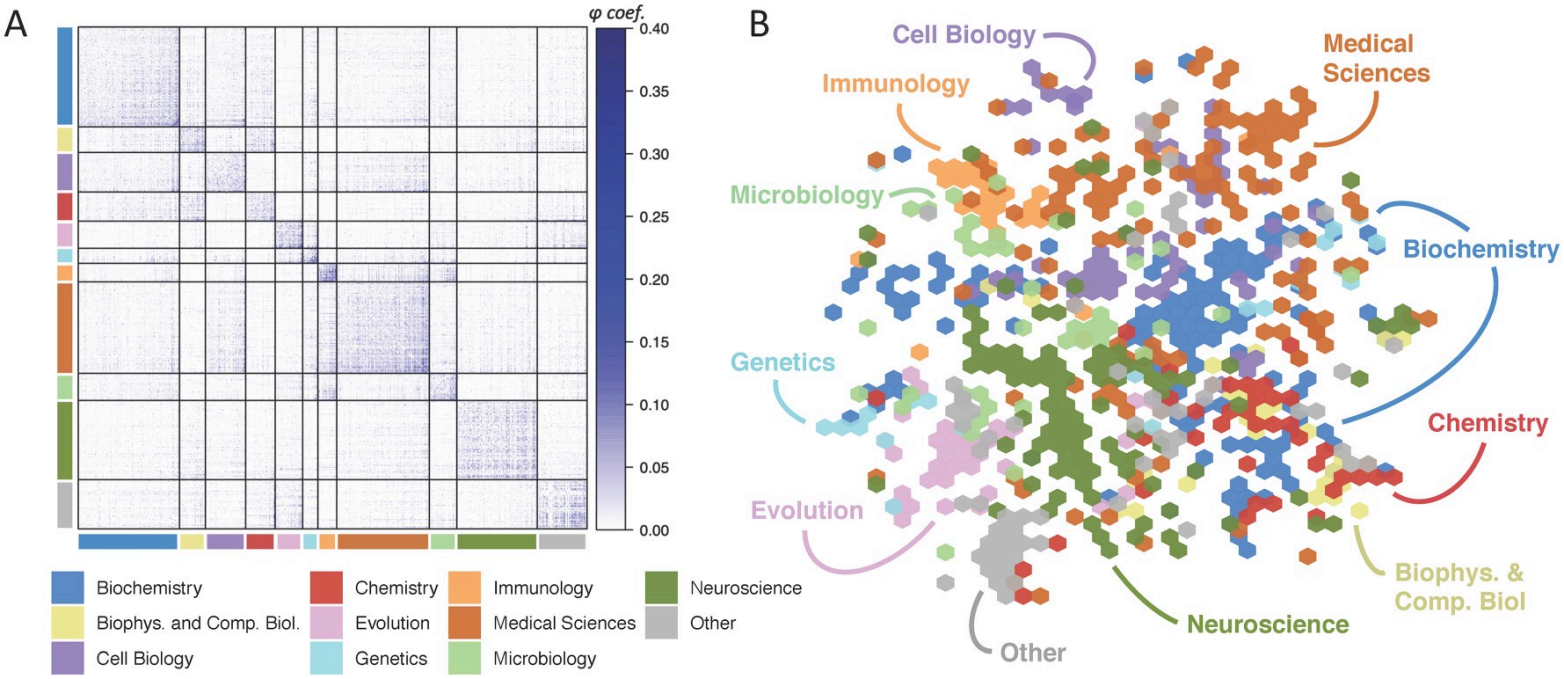
Architecture of the topic network. Nodes (*N* = 1000) reflect research topics and edges (*E* = 173, 309) reflect the extent of co-occurrence in abstracts and keyword sections. *(A)* The adjacency matrix sorted by topics’ most commonly associated article classification. *(B)* Visualization of the topic landscape using t-SNE [28], a method that places datapoints on a two-dimensional map based on their similarity. Nodes are colored by classification; “other” includes biophysics, developmental biology, ecology, environmental sciences, plant biology, and sustainability science.

To create the network, we drew potential topics from the *keywords* section of each article to allow for multi-word phrases. We determined the prevalence of each potential topic by finding the proportion of articles in which the word or phrase was contained in either the abstract or the keywords section. Based on this prevalence score, we identified the 1000 most common topics and represented each as a node in the network (See Methods for details). We then assigned a disciplinary classification to each topic, given by the most common classification among the articles in which the topic appeared (Fig 1).

Edges of the network represented the co-occurrence of topic *i* and topic *j* within abstracts, quantified by the *ϕ* coefficient of association for binary variables [24], given by,

$$\begin{array}{c} \phi_{ij}=\frac{A_{i\cap j}A_{i^{\prime }\cap j^{\prime }}-A_{i^{\prime }\cap j}A_{i\cap j^{\prime }}}{A_{i}A_{i^{\prime }}A_{j}A_{j^{\prime }}}, \end{array}$$ (1)

where *A*_*i*∩*j*_ gives the number of articles containing both topics *i* and *j*, *A*_*i*∩*j*′_ gives the number of articles containing topic *i* but not topic *j*, and *A*_*i*_ gives the number of articles containing topic *i*. In this context, the *ϕ* coefficient represents the extent to which articles tend to discuss both topics or neither topic relative to the extent to which they discuss one topic without the other. Similar constructions of topic similarity have been used to effectively capture network effects in prior research [18, 21], and this measure also approximately resembles the inverse of methods previously used to calculate the similarity between articles [10, 19].

Negative correlations—comprising roughly 65% of edges—were removed to increase the interpretability of the links between topics. As a result, the magnitude of a positive connection represents the extent of the association between two topics within the literature, and an edge weight of zero represents a lack of a conceptual relationship between two topics. Additionally, negative correlations between topics had notably lower magnitude and less variability (range: [-0.10,0], interquartile range: 0.004) than positive correlations (range: [0,0.84], interquartile range: 0.011), potentially suggesting that they contained less meaningful information than the remaining positive edges. To ensure that the estimated community structure was not overly dependent on this choice, we performed sensitivity analyses in which all edge weights were maintained. The effects of this choice on the community structure of the network are shown in S1 Fig.

### Structure of the topic network

To understand the structure of the topic network, we calculated measures of interconnectedness (global efficiency) and local clustering (average clustering coefficient); see S1 Appendix for mathematical definitions. For comparison, we obtained null adjacency matrices using the Hirschberger-Qi-Steuer algorithm [25], which accounts for structural features that are inherent to correlation-based networks [26, 27]. We observed that the topic network had significantly lower global efficiency (*p* < 0.01) and higher average clustering (*p* < 0.01) than was observed in the null correlation networks, indicating locally dense, non-random connectivity. See S1 Table for robustness of results to variations in network size.

**Fig 2:**
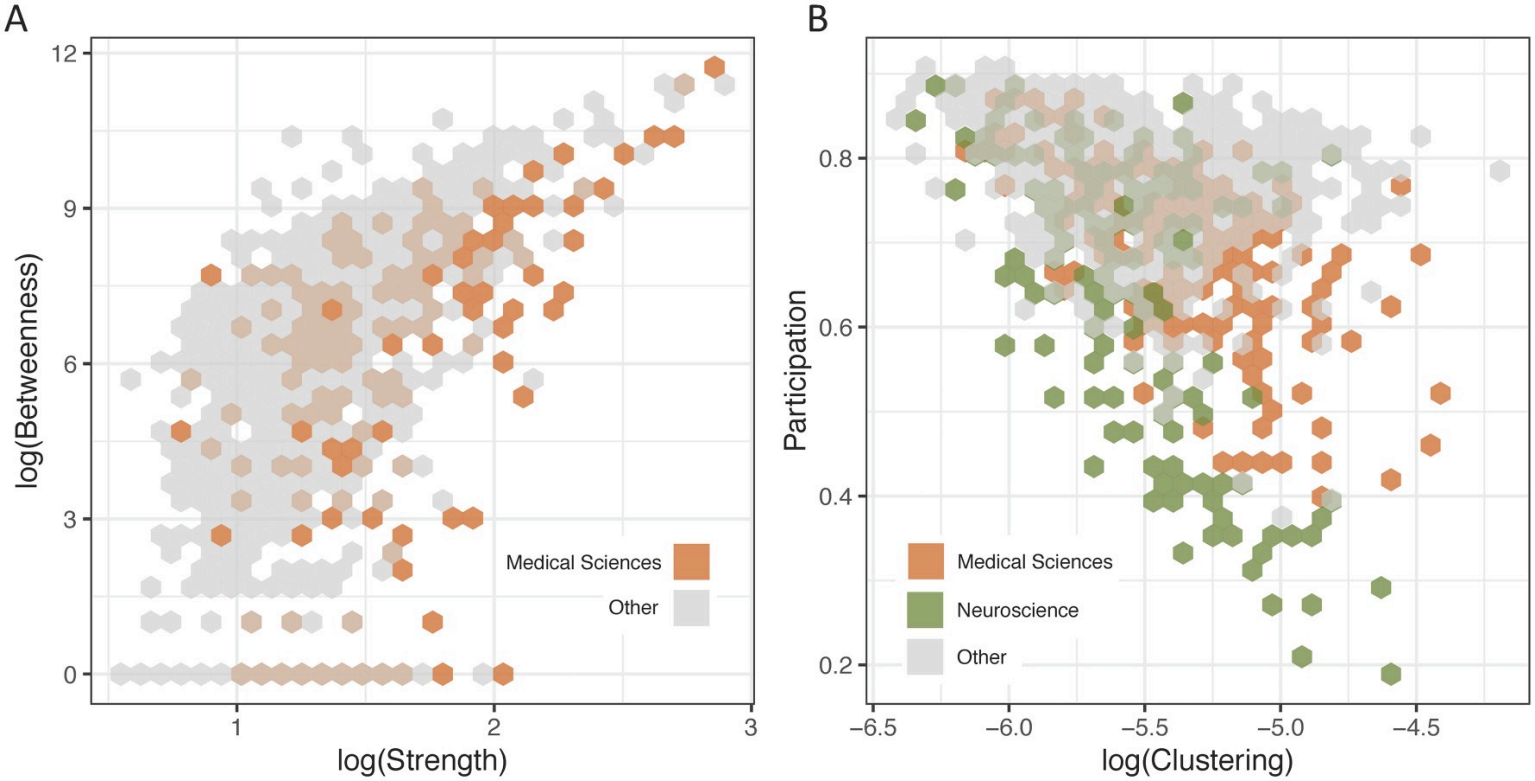
Structural relationships within the topic network. *(A)* Log-log relationship between topics’ strength and betweenness centrality. *(B)* Relationship between topics’ log-clustering coefficient and participation coefficient. Topics from notable disciplines are highlighted, and topics from other disciplines are gray.

To probe the local contributions of a topic to this overall structure, we examined each node’s general level of connectivity (degree, strength) and its role in bridging disparate regions of the network (betweenness centrality). Interestingly, we observed that betweenness centrality and degree were slightly negatively correlated (*ρ* = −0.14, *p* < 0.001), yet betweenness centrality and strength were moderately positively correlated (*ρ* = 0.55, *p* < 0.001; Fig 2A). These associations indicate that topics with high betweenness centrality tended to be those with strong connections to other topics, as opposed to those with many connections to other topics. Intuitively, this pattern suggests that high betweenness nodes in the topic network may more closely resemble “bridges” (nodes with a few strong/well-placed connections) than “hubs” (nodes with many connections), though the weak betweenness-degree relationship may indicate that both types are present.

The observed high local clustering and the presence of bridge nodes could be parsimoniously explained by the principle of small-worldness. To evaluate this possibility, we estimated the small-world propensity of the network [29]. Small-world propensity quantifies the extent to which a network shows similar local clustering to that of a lattice network (typically high), and similar average path length to that of a random network (typically low). This metric is similar to the commonly used small-world index, *σ* [30], but has been shown to be unbiased even in the context of networks with varying densities. Both measures broadly represent how well a network can be characterized as having both disparate clusters and high levels of between-cluster integration (see Methods for mathematical definition).

For the topic network, the observed small-world propensity was 0.57. This value was found to be significantly higher than would be expected of a random correlation-based network (*p* < 0.01). This result demonstrates that the relationships between topics have small-world properties, with more local clustering than would be expected of a random network and relatively efficient pathways between clusters. The presence of small-worldness then suggests that the topic network is naturally organized into a structure that may be well-suited for advancement within topic clusters and innovation between them.

While the presence of small-worldness in the network suggests separation between topic clusters, it remains an open question whether these clusters tend to fall along disciplinary lines. Using topics’ disciplinary classifications, we can quantify the trade-off between disciplinary diversity in a topic’s connections (participation coefficient) and local integration between a topic’s neighbors (clustering coefficient). Unsurprisingly, topics with more cross-disciplinary connections tended to show less local clustering (*ρ* = −0.46, *p* < 0.01; Fig 2B). Yet interestingly, topics generally had high participation throughout the network (*M* = 0.74, *SD* = 0.12), indicating that close connections between disciplines were common. Additionally, topics in neuroscience (*M* = 0.64, *SD* = 0.17) showed significantly lower participation than topics in other disciplines (*M* = 0.76, *SD* = 0.09; *t*_173_ = −8.78, *p* < 0.001).

### Community structure of the topic network

As the high participation across topics’ disciplinary classifications implies the presence of multidisciplinary relationships and clusters, we sought to identify the communities inherent in the data. Additionally, we set out to formally compare this data-driven partition to the partition arising from manually assigned disciplinary classifications.

First, we turned to the problem of identifying a natural partitioning of the topics based solely on the structure of the network, with no knowledge of the disciplinary classifications. We used a Louvain-like locally greedy algorithm [31, 32] to maximize the modularity, *Q*, of the network [33] (see Methods for details). The Louvain algorithm was chosen due to its efficient application within large networks, and comparably good performance relative to other “greedy” algorithms [34]. However, because of the stochastic nature of the Louvain algorithm, individual runs may return different local modularity maxima. To address this issue, we performed 100 optimizations, created an agreement matrix from the resulting partitions, and extracted a consensus partition to better represent the underlying community structure of the topics [35].

**Fig 3:**
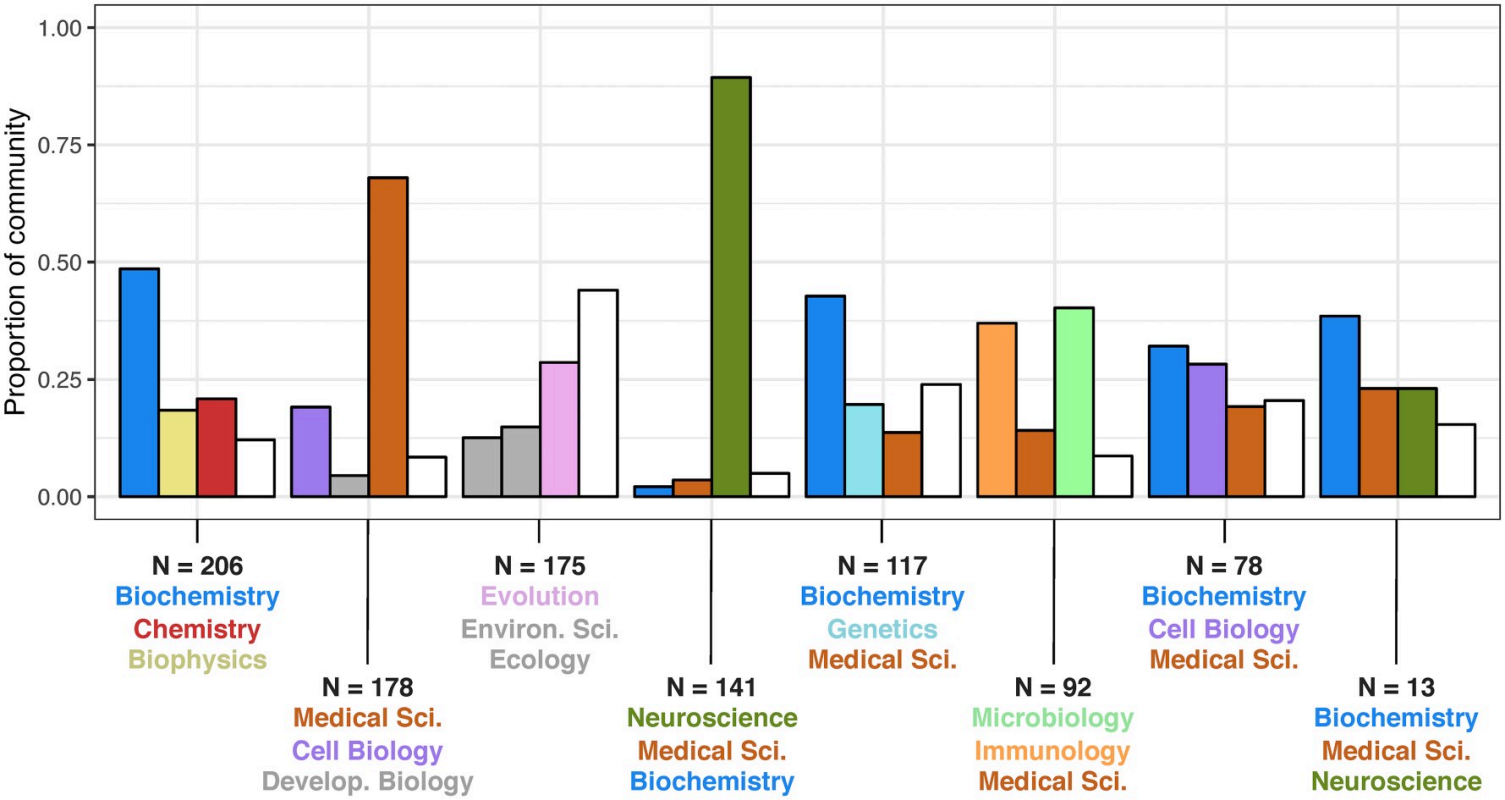
The topic network’s community structure. Data-driven communities are placed along the *x*-axis. The three most common topic disciplines contained within each community are presented in the graph and in the text below each community. The proportion of topics not contained within the three most common disciplines is shown in white for each community.

From this point forward, we refer to the consensus partition that resulted from the above process as the “data-driven partition”. It is important to note that various other empirical partitions could have been obtained from these data, so the name “data-driven partition” mostly serves to differentiate the consensus partition from the data-agnostic partition derived from article classifications. As compared to the 16 disciplinary communities implied by topics’ classifications, the data-driven partition yielded only eight distinct communities. Each community contained topics from various classifications, with relatively weak connections between communities (Fig 3 and S2 Table). It can be seen that communities are typically dominated by topics from two or three different disciplines. Two notable exceptions were a community made up almost entirely of neuroscience topics, which is consistent with the decreased participation in this discipline noted previously, and a community that was largely made up of medical sciences topics.

With the classification partition and data-driven partition in hand, we next sought to quantitatively compare the two. A natural way to formulate this comparison is to calculate the modularity for each partition to determine the extent of the separation between communities. As the data-driven partition was obtained by optimizing modularity and the classification partition was not, the data-driven partition will necessarily have a higher value on this metric. Yet the magnitude of the discrepancy is still informative, as it demonstrates the extent to which disciplinary classifications do or do not reflect a nearly optimal delineation of research topic clusters. We observed that the modularity value was 48% higher in the data-driven partition (*Q* = 0.37) than in the classification partition (*Q* = 0.25), indicating that the data-driven partition provided a more natural segregation into topic communities. Notably, this effect holds across a range of *γ* values, as the number of communities in the data-driven partition is varied from 8 to 16 (S3 Table). This fact suggests that community size did not drive the observed difference in modularity.

As further confirmation of the data-driven partition’s characterization of the community structure, we considered the framework of the weighted stochastic block model (WSBM; e.g., [36]), which provides a complementary means of quantifying how well a partition fits the data. Though there are many formulations of the WSBM and methods for obtaining WSBM partitions from networks, the framework broadly assumes a community structure in which edge weights are drawn from block-specific distributions, yielding expected weights for each edge. To investigate which partition better characterized the edge weights between and within communities, we fit an exponential distribution to the edge weights in each within- or between-community block. This process approximated a simplified version of the WSBM, where edge weight distributions were estimated from a fixed partition, as opposed to an optimal partition being estimated from the observed weights. We then calculated the squared difference between the observed edge weights and expected edge weights under the fit distributions. For each edge, this yielded a difference between the observed weight and the expected weight under the data-driven partition, $d_{ij}^{l}$, and another difference between the observed weight and the expected weight under the classification partition, $d_{ij}^{c}$. We applied a paired Wilcoxon signed rank test to these values to determine whether observed edge weights tended to be closer to one partition’s expected edge weights than the other’s. This test revealed that observed weights deviated from expected weights significantly more under the classification partition than the data-driven partition (*p* < 0.0001). Notably, this effect also holds across *γ* values and community sizes (S3 Table). Together, these findings indicate that the data-driven partition yields both stronger community separation and greater edge weight consistency within intracommunity and intercommunity blocks.

### Temporal changes in network structure

While the static structure of the topic network is important, it does not provide insight into whether and how the landscape of scientific inquiry might change over time. To address this question, we created a dynamic network using a 12-month sliding window with an 11-month overlap, tiling the period from January, 2000 to November, 2017. The 12-month width and 11-month overlap were chosen to induce smooth and gradual evolution of the network structure over time, as dramatic month-over-month changes in the relationships between scientific concepts are likely rare. Because some structural change would be expected due to random chance and patterns of journal publication over time, all measures were standardized relative to 100 iterations of a temporal null model where the order of article appearance was permuted uniformly at random. The null trajectories therefore represent change that would occur if topic prevalence and topic associations were stable over the full time period (see Methods for mathematical definition).

We first sought to test our hypothesis that the network would show strengthening connections between and within communities over time, consistent with increasing and changing patterns of collaboration [22, 23]. We tested for significant temporal changes in strength and small-world propensity by comparing the variance explained by the linear effect of year (R^2^) to distributions of R^2^ created from the trajectories of the 100 temporal null networks. Average strength (*R*^2^ = 0.75, *p* < 0.01) and small-world propensity (*R*^2^ = 0.25, *p* = 0.01) both showed significant positive linear trends over time; see S4 Table for consistent trends across network sizes). These results suggest that since 2000, associations between commonly covered scientific topics have grown stronger, and the extent to which these topics demonstrate high clustering and efficient pathways has increased as well.

Next, we sought to investigate whether the network’s interdisciplinarity showed a meaningful change over the time period under study. To accomplish this, we defined two novel measures of journal interdisciplinarity. The first, referred to as *unbalanced interdisciplinarity* (*ξ*_*U*_), is given by the overall difference between within-classification edge weights and between-classification edge weights across the network. It is defined as,

$$\begin{array}{c} \xi_{U}=\frac{\sum_{i,j}I\left\{c_{i}\neq c_{j}\right\}w_{ij}}{\sum_{i,j}I\left\{c_{i}\neq c_{j}\right\}}-\frac{\sum_{i,j}I\left\{c_{i}=c_{j}\right\}w_{ij}}{\sum_{i,j}I\left\{c_{i}=c_{j}\right\}}, \end{array}$$ (2)

where *c*_*i*_ is the classification of topic *i* within the set of classifications $\left\{C_{1},\ldots ,C_{N_{C}}\right\}$, *N*_*C*_ is the number of classifications in the network, and *w*_*ij*_ is the weight of the edge connecting topics *i* and *j*. Thus, this measure represents the overall tendency for between-classification relationships to be stronger or weaker than within-classification relationships. The measure increases as between-classification connection strength increases relative to within-classification connection strength overall.

The second measure, referred to as *balanced interdisciplinarity* (*ξ*_*B*_), is obtained by separately calculating the between-classification/within-classification difference for each classification, and then by averaging over all classifications within the network. It is defined as,

$$\begin{array}{c} \xi_{B}=\frac{1}{N_{C}}\sum_{k=1}^{N_{C}}[\frac{\sum_{i,j}I\left\{c_{i}=C_{k},c_{j}\neq C_{k}\right\}w_{ij}}{\sum_{i,j}I\left\{c_{i}=C_{k},c_{j}\neq C_{k}\right\}}-\frac{\sum_{i,j}I\left\{c_{i},c_{j}=C_{k}\right\}w_{ij}}{\sum_{i,j}I\left\{c_{i},c_{j}=C_{k}\right\}}]. \end{array}$$ (3)

Compared to *ξ*_*U*_, this measure facilitates the equal contribution of each classification to the overall score. Thus, whereas increases or decreases in *ξ*_*U*_ can be driven by one or two large disciplinary communities, *ξ*_*B*_ gives a picture of changes in interdisciplinarity that are occurring simultaneously across large and small fields.

**Fig 4:**
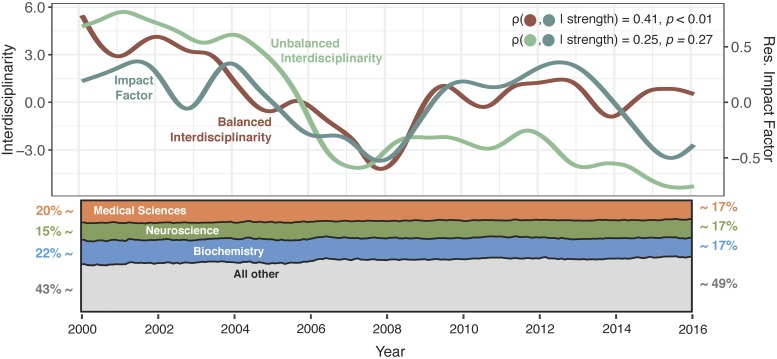
Temporal features of the dynamic topic network. *(Top row)* Temporal trajectories of unbalanced interdisciplinarity (green), balanced interdisciplinarity (maroon), and residual impact factor (blue) over time. *(Bottom row)* Temporal prevalence of topics from the three most common disciplines, relative to the prevalence of all other disciplines.

Unbalanced interdisciplinarity (*R*^2^ = 0.76, *p* < 0.01) showed a significant decrease over the time of study, while balanced interdisciplinarity did not (*R*^2^ = 0.11, *p* = 0.08) (Fig 4, top row). Since *ξ*_*U*_ captures shifts in the prevalence of different disciplines over time, it is possible that this pattern is reflecting a trend towards the publication of more siloed disciplines. Fig 4, bottom row, shows the trends in prevalence of the three most common topic classifications. While subtle, there does appear to be a gradual increase in the prevalence of smaller disciplines, which suggests that these smaller disciplines may tend to be less interdisciplinary than the larger disciplines. The lack of a similar decrease in *ξ*_*B*_ similarly suggests that the shifting balance of discipline prevalence was the driving factor behind the decrease in *ξ*_*U*_.

To begin understanding how interdisciplinary research is perceived, we compared the standardized trajectories of *ξ*_*U*_ and *ξ*_*B*_ to the trajectory of *PNAS*’s impact factor. We obtained yearly impact factors from 2000 to 2016 from the Web of Science, and fit a cubic spline to interpolate a smooth monthly trajectory. Interestingly, the number of articles published in a given time window explained 61% of the variation in impact factor; we therefore only considered the residuals. We calculated the partial correlation between standardized interdisciplinarity measures and impact factor after accounting for strength [37], and we compared the values to a null correlation distribution, obtained using the set of standardized trajectories drawn from the 100 temporal null networks described previously.

Unbalanced interdisciplinarity was not significantly associated with impact factor (*ρ* = 0.25, *p* = 0.27), but balanced interdisciplinarity showed a significant, positive partial correlation with impact factor (*ρ* = 0.41, *p* < 0.01) (Fig 4). These results suggest that fluctuations in interdisciplinarity that are shared across disciplines are associated with impact factor, but fluctuations that are driven by changing disciplinary prevalence may not be.

## Summary and discussion

Prior analyses of collaboration and citation networks have produced deep insights into the structures and relationships behind the production of scientific research [9, 11–14]. Yet little is known about the network structure of the scientific ideas themselves, or what features of this network might be most effective at facilitating innovation. Here, we sought to present a generalizable framework for understanding the structure that emerges from relationships between scientific topics. By applying this method to data from *PNAS*, we demonstrate the value of this framework for characterizing the structure of research topic networks, investigating whether topic communities tend to fit into disciplinary classifications, quantifying how the landscape of topics is changing over time, and determining whether a network’s interdisciplinarity may be related to the amount of engagement that its component research receives.

### Structure of the topic network

We constructed a network of research topics using seventeen years of *PNAS* articles, and found—unsurprisingly—that it had features uncharacteristic of a random correlation network. Specifically, the network had significantly higher clustering and lower efficiency than a random network, and showed patterns that revealed low-degree, high-strength bridge nodes that provide links within and between local clusters. The network also showed moderate to high small-worldness compared to what would be expected of a random correlation network. Both the graph statistical findings and the small-world classification are consistent with the networks described in studies of co-authorship and citation [13, 14], which would be expected to share many features with a network of research topics. As the structure of this network represents the structure of research from a single journal, it would be important for future work to determine whether these structural features hold for networks that examine research across multiple journals. If these features do hold, a possible next step would be to investigate the formation processes of these structures, potentially by examining the birth and growth of nodes and edges over time.

### Community structure of the topic network

Although community detection was referenced only as a future direction in seminal collaboration network analysis [12], the modular structure found in the topic network is consistent with the presence of communities in newer research on country-specific collaboration networks in both scientific and nonscientific fields [38, 39]. In this network, the empirical partition made up of multidisciplinary communities showed stronger separation between communities and provided a better fit to the within- and between-community edge weights than the partition arising from disciplinary classifications. Overall, the superior fit of the empirical communities compared to the classification-specific communities indicates that research published in *PNAS* is more interdisciplinary than the article classifications would suggest. One notable exception was a community made up almost exclusively of neuroscience topics (Fig 3), potentially reflecting neuroscience’s unique status as a field both popular enough to encompass many topics and young enough to remain largely insular. Yet the relative isolation of medical science topics complicates this interpretation, suggesting that further research is needed to understand the community-level mechanisms that drive or hamper interdisciplinarity.

### Temporal changes in network structure

Though the structure of the static network yielded valuable insights into the relationships between topics, the production of scientific research is far from static. Therefore, it was of great interest to examine temporal changes in the topic network. At the network scale, while generative evolution has been considered for authorship relationships [9, 40], the dynamic evolution of large-scale network properties has rarely been examined in the context of authorship or citation [41]. Here we found that both edge strength and small-worldness significantly increased over time. The strengthening of connections between seemingly distant research areas could reflect a convergence of the scientific landscape towards a more efficiently interconnected network of ideas. This would represent an interesting emergent property of the landscape, potentially arising from individual scientists consciously or unconsciously changing their behavior over time to perform more innovative work.

### Implications of interdisciplinarity

Despite the scientific push towards interdisciplinarity, and the prevalence of strong ties between disparate fields, the merits of interdisciplinarity are still widely debated [5, 6]. Proponents view interdisciplinary work as being crucial for “address[ing] the great questions of science” [42], while some skeptics instead believe that it too often represents “amateurism and intellectual voyeurism” [43]. In this study, we defined a novel measure of network interdisciplinarity, *ξ*_*B*_, and found it to be positively associated with *PNAS*’s impact factor.

Although this finding only speaks to work published in *PNAS*, within that context it suggests that bodies of work that are more interdisciplinary in nature may receive more engagement from the scientific community. Yet it remains unclear whether the increased engagement is reflective of the generation of more innovative scientific knowledge, or simply more effective dissemination of the knowledge across fields. In either case, this finding could reflect an important contribution to the discussion of interdisciplinary research, as previous research on the benefits of discipline-spanning has produced mixed results [6].

### Limitations

Validity and generalizability of the findings presented in this paper are limited by a few methodological considerations. First, seminal work has shown that fields of study differ significantly in the structures of their authorship and citation networks [12, 13]. Therefore it is likely that journals may also have meaningful differences in the structure and correlates of topic networks. Future work could expand the data source to include several top-tier journals within and across fields, and investigate the structural variability across journals, and the unique structural features of datasets that collapse across journals.

Additionally, the restriction of the dataset to keyword sections and abstracts may ignore potential information contained in introduction and discussion sections. However, it is plausible that topics mentioned in introduction and discussion areas may not be an accurate reflection of the topics truly covered in a given article, unlike those mentioned in the abstract and keyword sections. Future work could examine this assumption directly by implementing a manual rating system. Additionally, the current method does not account for synonyms, or for fields using different jargon to refer to identical concepts. Methods for discovering parallel scientific concepts being discussed independently within disparate disciplines would be immensely valuable within the framework of topic networks, and could potentially be pursued by assessing the existence and prevalence of cross-disciplinary topic pairs that share an abnormally high amount of neighbors within the network.

Finally, impact factor is widely considered to be an imperfect measure of scientific engagement with published research. Although warnings against impact factor’s use often highlight its inability to facilitate valid comparisons between journals in different fields or different countries [44, 45], within-journal changes over time are also incomplete and potentially subject to manipulation through editorial policies [46]. Future work could consider associations between network structure and other measures of scientific engagement and journal quality.

## Conclusion

In this study, we propose a topic network framework for investigating the emergent relational characteristics of concepts in scientific research, and apply it to articles published in *PNAS* since the year 2000. The topic network displayed small-world properties and interesting positive strength-betweenness/negative degree-betweenness associations, indicating the presence of tightly connected clusters and low-degree, high-strength nodes serving as conceptual bridges. Community detection showed that assigned classifications map poorly onto the underlying clusters, with a data-driven partition revealing the existence of multidisciplinary modules that contained topics from a variety of classifications. By investigating the temporal properties of the network, we found that both strength and small-worldness have been increasing over time. Interestingly, a novel measure of network interdisciplinarity was found to be positively associated with journal impact factor. Overall, this work demonstrates the value of network analysis in gaining insight into the structure of scientific knowledge, paints a picture of the surprisingly integrated nature of scientific ideas, and reveals a potentially important positive relationship between interdisciplinarity and scientific engagement.

## Materials and methods

### Data collection

We retrieved keywords and abstracts from 65,290 articles published in *PNAS* from the journal’s website using an in-house R script, and we used keyword sections to create a list of potential topics to be searched for in the abstracts. This technique was chosen over latent topic modeling, as it reflected scientists’ explicit opinions as to the words and phrases that constitute relevant scientific topics, and allowed for the incorporation of multi-word phrases.

### Full network construction

We calculated the prevalence of each potential topic by finding the ratio of abstracts or keyword sections containing the topic phrase to the total number of articles written in the time span of study. Thus, prevalence varied for the full network and the year-specific networks. We represented the 1000 most common topics in the given time span as network nodes; this value represented the approximate number at which the least prevalent words occurred often enough to produce a meaningful signal. Edges were given by the *ϕ* coefficient for binary association [24], and negative correlations were removed to improve interpretability and to allow for analysis of structural features that do not extend to signed networks.

### Temporal network construction

We created a dynamic network using a sliding window of ±6 months from a central month. Central months ranged from July, 2000 to May, 2017 such that data from January, 2000 to November, 2017 were included in the analyses. At each window, the 1000 most common topics were used as nodes. We made the choice of 1000 nodes for both the static and dynamic networks because it represented the highest number at which all topics selected in each window would occur more than five times. Thus, higher values would risk uninterpretable noise among low-prevalence topics, and lower values would sacrifice valuable information. A temporal null model was created to establish significance of temporal trends and correlations among network measures. This model can be formalized as follows. The observed edge weight between topic *i* and topic *j* in a specific temporal window is given by,

$$\begin{array}{c} \phi_{ij}^{\left(t\right)}=\frac{A_{i\cap j}^{\left(t\right)}A_{i^{\prime }\cap j^{\prime }}^{\left(t\right)}-A_{i^{\prime }\cap j}^{\left(t\right)}A_{i\cap j^{\prime }}^{\left(t\right)}}{A_{i}^{\left(t\right)}A_{i^{\prime }}^{\left(t\right)}A_{j}^{\left(t\right)}A_{j^{\prime }}^{\left(t\right)}}, \end{array}$$ (4)

where *A*^(*t*)^ is the set of articles that were published within a symmetric 12-month window around time *t*. By randomly sampling without replacement from the full set of articles, *A*, we created a reordered set of articles, *B*. We then assigned the dates from *A* to the reordered articles within *B*, and obtained *B*^(t*)^, which represents a random sample of articles that is the same size as *A*^(*t*)^. Null edge weights, $\phi_{ij}^{\left(\right)}$, are then given by,

$$\begin{array}{c} \phi_{ij}^{\left(t*\right)}=\frac{B_{i\cap j}^{\left(t*\right)}B_{i^{\prime }\cap j^{\prime }}^{\left(t*\right)}-B_{i^{\prime }\cap j}^{\left(t*\right)}B_{i\cap j^{\prime }}^{\left(t*\right)}}{B_{i}^{\left(t*\right)}B_{i^{\prime }}^{\left(t*\right)}B_{j}^{\left(t*\right)}B_{j^{\prime }}^{\left(t*\right)}}, \end{array}$$ (5)

Then for a given network measure, *θ*(*ϕ*^(*t*)^), which is a function of the adjacency matrix at time *t*, we can obtain a null measure, *θ*(*ϕ*^(t*)^). By applying this technique at every point *t*, a null temporal trajectory can be obtained, and null distributions of specific functions of these trajectories can be estimated.

### Community detection

For both the static and the dynamic networks, we performed community detection using an iterative generalized Louvain-like locally greedy algorithm to maximize a common modularity quality function [31, 32]. The modularity, *Q*, of a network intuitively represents the extent of separation between nodes in different groups [47]. It quantifies how well the network can be separated into non-overlapping communities, with many (or strong) within-group connections and few (or weak) between-group connections. For a network containing only positive weights, the modularity can be defined as follows:

$$\begin{array}{c} Q^{w}=\frac{1}{l^{w}}\sum_{i,j\in N}[w_{ij}-\frac{s_{i}s_{j}}{l^{w}}]\delta_{m_{i}m_{j}}, \end{array}$$ (6)

and for a signed network, the modularity can be defined as follows [48]:

$$\begin{array}{c} Q_{s}^{w}=\frac{1}{l_{+}^{w}+l_{-}^{w}}\sum_{i,j\in N}[w_{ij}-\frac{s_{i}^{+}s_{j}^{+}}{l_{+}^{w}}+\frac{s_{i}^{-}s_{j}^{-}}{l_{-}^{w}}]\delta_{m_{i}m_{j}}, \end{array}$$ (7)

where *l*^*w*^ is twice the sum of all of the weights in the network, $l_{+}^{w}$ is twice the sum of all of the positive weights in the network, $l_{-}^{w}$ is the sum of all of the negative weights in the network, $s_{i}^{+}$ is the strength of a node’s positive edges, $s_{i}^{-}$ is the strength of a node’s negative edges, and $\delta_{m_{i}m_{j}}$ is 1 if *i* = *j* and 0 otherwise. Note that the results of maximizing a signed modularity can be difficult to interpret [49], but in the current study the approach was used solely as a sensitivity analysis to investigate the effect of the decision to remove negative edges (see S1 Fig).

The Louvain-like community detection technique that was used in this study (GenLouvain; [32]) works by stochastic optimization of the quality index value *Q*, in which nodes are reassigned until no reassignment can improve *Q*, and then by iterating this optimization until convergence to an output partition. While this iteration helps somewhat with addressing the issue of near degeneracy [50] in the modularity landscape, the stochastic nature of the Louvain algorithm makes even iterated applications fall subject to local maxima. In an attempt to find a more empirical partition, we performed 100 separate iterations of the GenLouvain algorithm, created an agreement matrix from the 100 resulting partitions, and extracted a consensus partition from the agreement matrix according to the method described by Lancichinetti and Fortunato [35]. Repeated runs of this consensus procedure obtained an average pairwise Jaccard similarity of 0.96, showing strong consistency relative to individual partitions (average similarity = 0.74), and only small improvements over consensus procedures that used 40 partitions (average similarity = 0.93) and 70 partitions (average similarity = 0.95).

Additionally, to ensure that differences between the data-driven partition and the classification-based partition were not simply the result of differences in the scale of the communities, the free parameter used for modularity optimization, *γ*, was selected by maximizing the Jaccard similarity [51] between the two partitions. At steps of size 0.1 within 0 < *γ* ≤ 2 (after which Jaccard similarity was found to be strictly decreasing) the Louvain algorithm was performed 100 times, and the average Jaccard similarity at that *γ* value was calculated. The optimal *γ* was then taken to be the value at which similarity between the community-detection partition and the classification-based partition was highest (S2 Fig). This value was found to be *γ* = 1.2. Although more empirical methods for selecting *γ* have been proposed [52, 53], our method was chosen due to our focus on comparing derived communities to classification communities. Studies with other priorities for community detection may wish to utilize alternative methods for choosing *γ*.

## Supporting information

S1 Appendix Detailed description of data collection and network analysis techniques. (PDF)

S1 Table Effect of network size on the results for the full network. Rows represent different network level measures reported in the full text, columns represent their values and statistical significance for different choices of network size. Note: * = *p* < 0.05, ** = *p* < 0.01.
(PDF)

S2 Table Classification composition of empirically obtained topic communities. Rows represent the eight communities, and columns give the three most common classifications for the topics contained within each community.
(PDF)

S3 Table Effect of the number of communities on features of the empirical partition. Rows represent partitions with between 9 and 16 communities. Columns represent the extent to which the partitions demonstrate modular structure, contain disciplinary communities, and better explain edge weights compared to the classification partition.
(PDF)

S4 Table Effect of network size on the linear trajectories of the temporal network. Rows represent the linear change over time for various null-standardized measures of the temporal network. Columns represent the estimates and statistical significance for different choices of network size. Note: * = *p* < 0.05, ** = *p* < 0.01.
(PDF)

S5 Table Effect of network size on the impact factor correlations of the temporal network. Rows represent the correlations with *PNAS*’s impact factor for various measures of the temporal network. Columns represent their values and statistical significance for different choices of network size. Note: * = *p* < 0.05, ** = *p* < 0.01.
(PDF)

S1 Fig Visualization of the consistency, using Jaccard similarity, of the empirical community structure both (i) across sizes, and (ii) with or without negative edge weights. Community structure was consistent across sizes, and was reasonably consistent between positive weighted networks and positive-and-negative weighted networks.
(TIFF)

S2 Fig Visualization of the Jaccard similarity between the empirical community structure and the assigned topic classifications. Jaccard similarities are plotted for a range of *γ* values, demonstrating the procedure for optimizing Jaccard similarity over *γ* that was used when performing community detection. These values are shown for three different choices of network size.
(TIFF)

## References

[1] Wang D, Song C, Barabási AL. Quantifying long-term scientific impact. *Science*, 342(6154):127–132, 10 2013 ISSN 0036-8075, 1095-9203. URL http://www.sciencemag.org/lookup/doi/10.1126/science.1237825. 24092745 10.1126/science.1237825

[2] Acuna DE, Allesina S, Kording KP. Predicting scientific success: Future impact. *Nature*, 489(7415):201–202, 9 2012 ISSN 0028-0836, 1476-4687. URL http://www.nature.com/articles/489201a. 22972278 10.1038/489201a PMC3770471

[3] Erosheva E, Fienberg S, Lafferty J. Mixed-membership models of scientific publications. *Proceedings of the National Academy of Sciences*, 101(Supplement 1):5220–5227, 4 2004 ISSN 0027-8424, 1091-6490. URL http://www.pnas.org/cgi/doi/10.1073/pnas.0307760101. 10.1073/pnas.0307760101 PMC387299 15020766

[4] Airoldi EM, Erosheva EA, Fienberg SE, Joutard C, Love T, Shringarpure S. Reconceptualizing the classification of PNAS articles. *Proceedings of the National Academy of Sciences*, 107(49):20899–20904, 12 2010 ISSN 0027-8424, 1091-6490. URL http://www.pnas.org/cgi/doi/10.1073/pnas.1013452107. 10.1073/pnas.1013452107 PMC3000298 21078953

[5] Rhoten D, Parker A. Risks and rewards of an interdisciplinary research path. *Science*, 306(5704):2046–2046, 12 2004 ISSN 0036-8075, 1095-9203. URL http://www.sciencemag.org/cgi/doi/10.1126/science.1103628. 15604393 10.1126/science.1103628

[6] Jacobs JA, Frickel S. Interdisciplinarity: A critical assessment. *Annual Review of Sociology*, 35(1):43–65, 8 2009 ISSN 0360-0572, 1545-2115. URL http://www.annualreviews.org/doi/10.1146/annurev-soc-070308-115954.

[7] Levitt JM, Thelwall M. Is multidisciplinary research more highly cited? A macrolevel study. *Journal of the American Society for Information Science and Technology*, 59(12):1973–1984, 10 2008 ISSN 15322882, 15322890. URL http://doi.wiley.com/10.1002/asi.20914.

[8] Leahey E, Moody J. Sociological innovation through subfield integration. *Social Currents*, 1(3):228–256, 10 2014 ISSN 2329-4965, 2329-4973. URL http://journals.sagepub.com/doi/10.1177/2329496514540131.

[9] Newman MEJ. Clustering and preferential attachment in growing networks. *Physical Review E*, 64(2), 7 2001a ISSN 1063-651X, 1095-3787. URL https://link.aps.org/doi/10.1103/PhysRevE.64.025102. 10.1103/PhysRevE.64.025102 11497639

[10] Borrett SR, Moody J, Edelmann A. The rise of Network Ecology: Maps of the topic diversity and scientific collaboration. *Ecological Modelling*, 293:111–127, 12 2014 ISSN 03043800. URL http://linkinghub.elsevier.com/retrieve/pii/S0304380014001136.

[11] Chen C. Searching for intellectual turning points: Progressive knowledge domain visualization. *Proceedings of the National Academy of Sciences*, 101(Supplement 1):5303–5310, 4 2004 ISSN 0027-8424, 1091-6490. URL http://www.pnas.org/cgi/doi/10.1073/pnas.0307513100. 10.1073/pnas.0307513100 PMC387312 14724295

[12] Newman MEJ. Coauthorship networks and patterns of scientific collaboration. *Proceedings of the National Academy of Sciences*, 101(Supplement 1):5200–5205, 4 2004a ISSN 0027-8424, 1091-6490. URL http://www.pnas.org/cgi/doi/10.1073/pnas.0307545100. 10.1073/pnas.0307545100 PMC387296 14745042

[13] Newman MEJ. The structure of scientific collaboration networks. *Proceedings of the National Academy of Sciences*, 98(2):404–409, 1 2001b ISSN 00278424. URL https://www.pnas.org/content/98/2/404. 10.1073/pnas.021544898 PMC14598 11149952

[14] Wallace ML, Larivière V, Gingras Y. A small world of citations? The influence of collaboration networks on citation practices. *PLoS ONE*, 7(3):e33339, 3 2012 ISSN 1932-6203. URL http://dx.plos.org/10.1371/journal.pone.0033339. 22413016 10.1371/journal.pone.0033339 PMC3296690

[15] Radhakrishnan S, Erbis S, Isaacs JA, Kamarthi S. Novel keyword co-occurrence network-based methods to foster systematic reviews of scientific literature. *PLOS ONE*, 12(3):e0172778, 3 2017 ISSN 1932-6203. URL http://dx.plos.org/10.1371/journal.pone.0172778. 28328983 10.1371/journal.pone.0172778 PMC5362196

[16] Zhang J, Xie J, Hou W, Tu X, Xu J, Song F, et al Mapping the knowledge structure of research on patient adherence: Knowledge domain visualization based co-word analysis and social network analysis. *PLoS ONE*, 7(4):e34497, 4 2012 ISSN 1932-6203. URL http://dx.plos.org/10.1371/journal.pone.0034497. 22496819 10.1371/journal.pone.0034497 PMC3320627

[17] Peters HPF, van Raan AFJ. Co-word-based science maps of chemical engineering. Part I: Representations by direct multidimensional scaling. *Research Policy*, 22(1):23–45, 2 1993 ISSN 00487333. URL http://linkinghub.elsevier.com/retrieve/pii/004873339390031C.

[18] Beam E, Appelbaum LG, Jack J, Moody J, Huettel SA. Mapping the semantic structure of cognitive neuroscience. *Journal of Cognitive Neuroscience*, 26(9):1949–1965, 9 2014 ISSN 0898-929X, 1530-8898. URL http://www.mitpressjournals.org/doi/10.1162/jocn_a_00604. 24666126 10.1162/jocn_a_00604

[19] Moody J, Light R. A view from above: The evolving sociological landscape. *The American Sociologist*, 37(2):67–86, 6 2006 ISSN 0003-1232, 1936-4784. URL http://link.springer.com/10.1007/s12108-006-1006-8.

[20] Frangi AF, Taylor ZA, Gooya A. Precision Imaging: More descriptive, predictive and integrative imaging. *Medical Image Analysis*, 33:27–32, 10 2016 ISSN 13618415. URL http://linkinghub.elsevier.com/retrieve/pii/S1361841516301049. 27373145 10.1016/j.media.2016.06.024

[21] Dworkin JD, Shinohara RT, Bassett DS. The landscape of NeuroImage-ing research. *NeuroImage*, 183:872–883, 12 2018 ISSN 10538119. URL https://linkinghub.elsevier.com/retrieve/pii/S105381191830778X. 30195054 10.1016/j.neuroimage.2018.09.005 PMC6197920

[22] Hoekman J, Frenken K, Tijssen RJW. Research collaboration at a distance: Changing spatial patterns of scientific collaboration within Europe. *Research Policy*, 39(5):662–673, 6 2010 ISSN 00487333. URL http://linkinghub.elsevier.com/retrieve/pii/S0048733310000260.

[23] Huang J, Zhuang Z, Li J, Giles CL. Collaboration over time: Characterizing and modeling network evolution. page 107. ACM Press, 2008. URL http://portal.acm.org/citation.cfm?doid=1341531.1341548.

[24] Davenport EC, El-Sanhurry NA. Phi/Phimax: Review and synthesis. *Educational and Psychological Measurement*, 51(4):821–828, 12 1991 ISSN 0013-1644, 1552-3888. URL http://journals.sagepub.com/doi/10.1177/001316449105100403.

[25] Hirschberger M, Qi Y, Steuer RE. Randomly generating portfolio-selection covariance matrices with specified distributional characteristics. *European Journal of Operational Research*, 177:(3) 1610–1625, 3 2007 ISSN 03772217. URL http://linkinghub.elsevier.com/retrieve/pii/S0377221705006600.

[26] Zalesky A, Fornito A, Bullmore E. On the use of correlation as a measure of network connectivity. *NeuroImage*, 60:(4) 2096–2106, 5 2012 ISSN 10538119. URL https://linkinghub.elsevier.com/retrieve/pii/S1053811912001784. 22343126 10.1016/j.neuroimage.2012.02.001

[27] Masuda N, Sakaki M, Ezaki T, Watanabe T. Clustering coefficients for correlation networks. *Fronteirs in Neuroinformatics*, 12: 3 2018 ISSN 1662-5196. URL http://journal.frontiersin.org/article/10.3389/fninf.2018.00007/full. 10.3389/fninf.2018.00007 PMC5863042 29599714

[28] van der Maaten L, Hinton GE. Visualizing high-dimensional data using t-sne. *Journal of Machine Learning Research*, 9:2579–2605, 1 2008.

[29] Muldoon SF, Bridgeford EW, Bassett DS. Small-world propensity and weighted brain networks. *Scientific Reports*, 6(1), 4 2016 ISSN 2045-2322. URL http://www.nature.com/articles/srep22057. 10.1038/srep22057 PMC4766852 26912196

[30] Watts DJ, Strogatz SH. Collective dynamics of’small-world’ networks. *Nature*, 393(6684):440–442, 6 1998 ISSN 0028-0836. 10.1038/30918 9623998

[31] Blondel VD, Guillaume JL, Lambiotte R, Lefebvre E. Fast unfolding of communities in large networks. *Journal of Statistical Mechanics: Theory and Experiment*, 2008(10), 10 2008 ISSN 1742-5468. URL http://stacks.iop.org/1742-5468/2008/i=10/a=P10008.

[32] Jeub LGS, Bazzi M, Jutla IS, Mucha PJ. A generalized Louvain method for community detection implemented in MATLAB, 2011. URL http://netwiki.amath.unc.edu/GenLouvain.

[33] Newman MEJ. Modularity and community structure in networks. *Proc. Natl. Acad. Sci. U.S.A*., 103(23):8577–8582, 2006 10.1073/pnas.0601602103 16723398 PMC1482622

[34] Fortunato S. Community detection in graphs. *Physics Reports*, 486(3-5):75–174, 2 2010 ISSN 03701573. URL https://linkinghub.elsevier.com/retrieve/pii/S0370157309002841.

[35] Lancichinetti A, Fortunato S. Consensus clustering in complex networks. *Scientific Reports*, 2(1), 12 2012 ISSN 2045-2322. URL http://www.nature.com/articles/srep00336. 22468223 10.1038/srep00336 PMC3313482

[36] Aicher C, Jacobs AZ, Clauset A. Learning latent block structure in weighted networks. *Journal of Complex Networks*, 3(2):221–248, 6 2015 ISSN 2051-1310, 2051-1329. URL https://academic.oup.com/comnet/article-lookup/doi/10.1093/comnet/cnu026.

[37] van Wijk BCM, Stam CJ, Daffertshofer A. Comparing brain networks of different size and connectivity density using graph theory. *PLoS ONE*, 5(10):e13701, 10 2010 ISSN 1932-6203. URL http://dx.plos.org/10.1371/journal.pone.0013701. 21060892 10.1371/journal.pone.0013701 PMC2965659

[38] Lužar B, Levnajić Z, Povh J, Perc M. Community structure and the evolution of interdisciplinarity in Slovenia’s scientific collaboration network. *PLoS ONE*, 9(4):e94429, 4 2014 ISSN 1932-6203. URL http://dx.plos.org/10.1371/journal.pone.0094429. 24728345 10.1371/journal.pone.0094429 PMC3984150

[39] Battiston F, Iacovacci J, Nicosia V, Bianconi G, Latora V. Emergence of multiplex communities in collaboration networks. *PLOS ONE*, 11(1):e0147451, 1 2016 ISSN 1932-6203. URL http://dx.plos.org/10.1371/journal.pone.0147451. 26815700 10.1371/journal.pone.0147451 PMC4731389

[40] Wagner CS, Leydesdorff L. Network structure, self-organization, and the growth of international collaboration in science. *Research Policy*, 34(10):1608–1618, 12 2005 ISSN 00487333. URL http://linkinghub.elsevier.com/retrieve/pii/S0048733305001745.

[41] Kronegger L, Ferligoj A, Doreian P. On the dynamics of national scientific systems. *Quality & Quantity*, 45(5):989–1015, 8 2011 ISSN 0033-5177, 1573-7845. URL http://link.springer.com/10.1007/s11135-011-9484-3.

[42] *Facilitating interdisciplinary research*. National Academies Press, Washington, D.C., 4 2004 ISBN 978-0-309-09435-1. URL http://www.nap.edu/catalog/11153.

[43] Davis LJ. A grand unified theory of interdisciplinarity. *Chronicles of Higher Education*, 53(40):B9, 2007.

[44] Bordons M, Fernández MT, Gómez I. Advantages and limitations in the use of impact factor measures for the assessment of research performance. *Scientometrics*, 53:195–206, 2 2002 10.1023/A:1014800407876

[45] Kurmis AP. Understanding the limitations of the journal impact factor. *The Journal of Bone and Joint Surgery*. American Volume, 85-A(12):2449–2454, 12 2003 ISSN 0021-9355. 10.2106/00004623-200312000-00028 14668520

[46] Chew M, Villanueva EV, Van Der Weyden MB. Life and times of the impact factor: retrospective analysis of trends for seven medical journals (1994-2005) and their Editors’ views. *Journal of the Royal Society of Medicine*, 100(3):142–150, 3 2007 ISSN 0141-0768, 0141-0768. URL http://www.jrsm.org/cgi/doi/10.1258/jrsm.100.3.142. 17339310 10.1258/jrsm.100.3.142 PMC1809163

[47] Newman MEJ. Analysis of weighted networks. *Physical Review E*, 70(5), 11 2004b ISSN 1539-3755, 1550-2376. URL https://link.aps.org/doi/10.1103/PhysRevE.70.056131. 10.1103/PhysRevE.70.056131 15600716

[48] Gómez S, Jensen P, Arenas A. Analysis of community structure in networks of correlated data. *Physical Review E*, 80(1), 7 2009 ISSN 1539-3755, 1550-2376. URL https://link.aps.org/doi/10.1103/PhysRevE.80.016114. 10.1103/PhysRevE.80.016114 19658781

[49] Bazzi M, Porter MA, Williams S, McDonald M, Fenn DJ, Howison SD. Community detection in temporal multilayer networks, with an application to correlation networks. *Multiscale Modeling & Simulation*, 14(1):1–41, 1 2016 ISSN 1540-3459, 1540-3467. URL http://epubs.siam.org/doi/10.1137/15M1009615.

[50] Good BH, de Montjoye YA, Clauset A. Performance of modularity maximization in practical contexts. *Physical Review E*, 81(4), 4 2010 ISSN 1539-3755, 1550-2376. URL https://link.aps.org/doi/10.1103/PhysRevE.81.046106. 10.1103/PhysRevE.81.046106 20481785

[51] Steen M, Hayasaka S, Joyce K, Laurienti P. Assessing the consistency of community structure in complex networks. *Physical Review E*, 84(1), 7 2011 ISSN 1539-3755, 1550-2376. URL https://link.aps.org/doi/10.1103/PhysRevE.84.016111. 10.1103/PhysRevE.84.016111 PMC3292265 21867261

[52] Jeub LGS, Sporns O, Fortunato S. Multiresolution consensus clustering in networks. *Scientific Reports*, 8(1), 12 2018 ISSN 2045-2322. URL http://www.nature.com/articles/s41598-018-21352-7. 29459635 10.1038/s41598-018-21352-7 PMC5818657

[53] Newman MEJ. Equivalence between modularity optimization and maximum likelihood methods for community detection. *Physical Review E*, 94(5), 11 2016 ISSN 2470-0045, 2470-0053. URL https://link.aps.org/doi/10.1103/PhysRevE.94.052315. 10.1103/PhysRevE.94.052315 27967199

