# Supplementary material for: The emergent integrated network structure of scientific research
Source: PLoS One. 2019 Apr 30;14(4):e0216146. doi: 10.1371/journal.pone.0216146 (PMC6490937; doi:10.1371/journal.pone.0216146)
Supplement: S1 Appendix — (PDF) [file pone.0216146.s001.pdf]

S1 Appendix

Data analysis

Topic networks and temporal null networks were created, visualized, and analyzed in the R statistical environment [1] using the iGraph package [2]. Benchmark random networks were generated using the Brain Connectivity Toolbox in MATLAB [3], but were analyzed using the iGraph package. Community detection was carried out in MATLAB using the GenLouvain toolbox [4]. Correlation coefficients and probability values were obtained using the Hmisc package in R [5].

Network Measures

Here we provide a brief description of the more common network measures used in this study.

The **degree** of a node is the number of edges, regardless of weight, connected to the node [3]. Degree then represents one aspect of the node's importance, measured by the number of neighbors it has in the network. It is defined as follows:

$$k_i = \sum_{j \in N} a_{ij}, \tag{1}$$

where N is the set of all nodes in the network, and a_{ij} is 1 if nodes i and j are connected by an edge and 0 if not.

The **strength** of a node is the sum of the weights of all edges connected to the node [3]. This measure is similar to degree in that it sums a node's connecting edges, but strength additionally allows for edges of varying weights. It is defined as follows:

$$s_i = \sum_{j \in N} w_{ij}, \tag{2}$$

where w_{ij} is the weight of the edge between nodes i and j if they are connected and 0 if not.

The **betweenness centrality** of a node is the proportion of all shortest paths within the network that pass through the given node [6]. Betweenness centrality represents the extent to which a specific node functions as a bridge between nodes in disparate parts of the network. It is defined as follows:

$$b_i = \frac{1}{(n-1)(n-2)} \sum_{h,j \in N; h \neq j, h \neq i, j \neq i} \frac{\rho_{hj}^{(i)}}{\rho_{hj}}, \tag{3}$$

where ρ_{hj} is the number of shortest weighted paths between h and j, $\rho_{hj}^{(i)}$ is the number of shortest weighted paths between h and j that pass through node i, and n is the number of nodes in the graph.

The **participation coefficient** of a node is the extent to which it is strongly associated with nodes in modules or communities other than its own [7]. Participation falls between 0 and 1, with higher values representing more diversity within a node's connections. It is defined as follows:

$$y_i^w = 1 - \sum_{m \in M} \left(\frac{s_i(m)}{s_i} \right)^2,$$

where M is the set of modules and $s_i(m)$ is sum of edge weights from node i to other nodes in module m.

The **clustering coefficient** of a node can be defined as the probability that two of its adjacent nodes are connected to each other. A node's clustering coefficient then represents the amount of interconnectedness in a node's local neighborhood. The version used in the current study is a measure of transitivity, as given by Barrat [8]. It is defined as follows:

$$t_i^w = \frac{1}{s_i(k_i - 1)} \sum_{h,j \in N} \frac{(w_{ij} + w_{ih})}{2} a_{ij} a_{ih} a_{hj}. \tag{4}$$

The **global efficiency** of a network can be defined as the average inverse shortest path length between any two nodes [9]. Global efficiency is often thought of as representing the amount of integration within and between disparate parts of the network. It is defined as follows:

$$E^w = \frac{1}{n} \sum_{i \in N} \frac{\sum_{j \in N; j \neq i} d_{ij}^{-1}}{n - 1}, \tag{5}$$

where d_{ij} is the shortest weighted path length between node i and node j, defined as the minimum distance required to traverse the graph from node i to node j. The algorithm for calculating this measure is described by Newman [10].

The **path length** of a network is the average shortest path length between all node pairs [11]. In many graphs, path length is inversely correlated with global efficiency, and is therefore often interpreted as representing an alternative measure of network integration. A version of the path length for a weighted network is as follows:

$$L = \frac{1}{n(n-1)} \sum_{i \neq j} d_{ij}. \tag{6}$$

The **small-world propensity** measures the extent to which a network is characterized by high levels of local clustering and low average path length [12]. It is defined as follows:

$$\Phi = 1 - \sqrt{\frac{\Delta_T^2 + \Delta_L^2}{2}}, \tag{7}$$

where

$$\Delta_T = \frac{T_{lattice} - T_{observed}}{T_{lattice} - T_{random}}, \tag{8}$$

and

$$\Delta_L = \frac{L_{observed} - L_{random}}{L_{lattice} - L_{random}}, \tag{9}$$

with T representing the network clustering (transitivity) coefficient, defined as the average node-specific t_i^w values.

The **stochastic block model** assumes a community structure in which between- and within-group connections occur with a specific probability (in the unweighted case) or an expected edge weight (in the weighted case). Unlike modularity, which characterizes a community structure with many (strong) connections within groups and few (weak) connections between groups, the stochastic block model characterizes a community structure with consistent connection patterns within and between groups. For the unweighted case [13], it is defined as follows:

$$P_{g,B}(A) = \prod_{i \neq j} B_{g_i g_j}^{a_{ij}} \left(1 - B_{g_i g_j}\right)^{(1-a_{ij})}, \tag{10}$$

where $g \in 1, ..., K^n$ is a vector of community memberships, assuming K distinct communities, and $B \in [0,1]^{KxK}$ is a matrix of community-wise edge probabilities.

For the exponential weighted framework used in the current study [14], the model is defined as follows:

$$P_{g,\Lambda}(A) = \prod_{i \neq j} \Lambda_{g_i g_j} e^{-\Lambda_{g_i g_j} w_{ij}}, \tag{11}$$

where $\Lambda \in [0, \infty)^{KxK}$ is a matrix of community-wise rate parameters.

References

1. R: A language and environment for statistical computing, 2015.
2. Csardi G, Nepusz T. The igraph software package for complex network research. *InterJournal*, Complex Systems:1695, 2006. URL `http://igraph.org`.
3. Rubinov M, Sporns O. Complex network measures of brain connectivity: Uses and interpretations. *NeuroImage*, 52(3):1059–1069, September 2010. ISSN 10538119. doi: 10.1016/j.neuroimage.2009.10.003. URL `http://linkinghub.elsevier.com/retrieve/pii/S105381190901074X`.
4. Jeub LGS, Bazzi M, Jutla IS, Mucha PJ. A generalized Louvain method for community detection implemented in MATLAB, 2011. URL `http://netwiki.amath.unc.edu/GenLouvain`.
5. Harrell Jr. FE, with contributions from Dupont C, and many others. *Hmisc: Harrell Miscellaneous*, 2016. URL `https://CRAN.R-project.org/package=Hmisc`. R package version 4.0-2.
6. Freeman LC. Centrality in social networks conceptual clarification. *Social Networks*, 1(3):215–239, January 1978. ISSN 03788733. doi: 10.1016/0378-8733(78)90021-7. URL `http://linkinghub.elsevier.com/retrieve/pii/0378873378900217`.
7. Guimerà R, Nunes Amaral LA. Cartography of complex networks: modules and universal roles. *Journal of Statistical Mechanics: Theory and Experiment*, 2005 (02):1–13, February 2005. doi: 10.1088/1742-5468/2005/02/P02001.
8. Barrat A, Barthelemy M, Pastor-Satorras R, Vespignani A. The architecture of complex weighted networks. *Proceedings of the National Academy of Sciences*, 101 (11):3747–3752, March 2004. ISSN 0027-8424, 1091-6490. doi: 10.1073/pnas.0400087101. URL `http://www.pnas.org/cgi/doi/10.1073/pnas.0400087101`.
9. Latora V, Marchiori M. Efficient behavior of small-world networks. *Physical Review Letters*, 87(19), October 2001. ISSN 0031-9007, 1079-7114. doi: 10.1103/PhysRevLett.87.198701. URL `https://link.aps.org/doi/10.1103/PhysRevLett.87.198701`.
10. Newman MEJ. Scientific collaboration networks. II. Shortest paths, weighted networks, and centrality. *Physical Review E*, 64(1), June 2001. ISSN 1063-651X, 1095-3787. doi: 10.1103/PhysRevE.64.016132. URL `https://link.aps.org/doi/10.1103/PhysRevE.64.016132`.
11. Watts DJ, Strogatz SH. Collective dynamics of 'small-world' networks. *Nature*, 393(6684):440–442, June 1998. ISSN 0028-0836. doi: 10.1038/30918.
12. Muldoon SF, Bridgeford EW, Bassett DS. Small-world propensity and weighted brain networks. *Scientific Reports*, 6(1), April 2016. ISSN 2045-2322. doi: 10.1038/srep22057. URL `http://www.nature.com/articles/srep22057`.
13. Wang YJ, Wong GY. Stochastic blockmodels for directed graphs. *Journal of the American Statistical Association*, 82(397):8–19, March 1987. ISSN 0162-1459, 1537-274X. doi: 10.1080/01621459.1987.10478385. URL `http://www.tandfonline.com/doi/abs/10.1080/01621459.1987.10478385`.

14. Aicher C, Jacobs AZ, Clauset A. Learning latent block structure in weighted networks. *Journal of Complex Networks*, 3(2):221–248, June 2015. ISSN 2051-1310, 2051-1329. doi: 10.1093/comnet/cnu026. URL `https://academic.oup.com/comnet/article-lookup/doi/10.1093/comnet/cnu026`.
