# Supplementary material for: The emergent integrated network structure of scientific research
Source: PLoS One. 2019 Apr 30;14(4):e0216146. doi: 10.1371/journal.pone.0216146 (PMC6490937; doi:10.1371/journal.pone.0216146)
Supplement: S1 Table — Rows represent different network level measures reported in the full text, columns represent their values and statistical significance for different choices of network size. Note: * = p < 0.05, ** = p < 0.01. (PDF) [file pone.0216146.s002.pdf]

Network Measure	N = 950	N = 1000	N = 1050
Small-world propensity (ϕ)	0.59**	0.58**	0.57**
Global efficiency (E^w)	0.029**	0.029**	0.028**
Average clustering (c^w)	0.004**	0.004**	0.004**
Betweenness - degree correlation (r)	-0.14**	-0.14**	-0.12**
Betweenness - strength correlation (r)	0.56**	0.55**	0.55**
Participation - clustering correlation (r)	-0.45**	-0.46**	-0.47**

S1 Table. Effect of network size on the results for the full network. Rows represent different network level measures reported in the full text, columns represent their values and statistical significance for different choices of network size. Note: * = $p < 0.05$, ** = $p < 0.01$.
