# Supplementary material for: The emergent integrated network structure of scientific research
Source: PLoS One. 2019 Apr 30;14(4):e0216146. doi: 10.1371/journal.pone.0216146 (PMC6490937; doi:10.1371/journal.pone.0216146)
Supplement: S2 Table — Rows represent the eight communities, and columns give the three most common classifications for the topics contained within each community. (PDF) [file pone.0216146.s003.pdf]

Group	# of topics	Primary topic classification	Secondary topic classification	Tertiary topic classification
1	206	Biochemistry (49%)	Chemistry (21%)	Biophysics and Comp. Biol (18%)
2	178	Medical Sciences (68%)	Cell Biology (19%)	Developmental Biology (4%)
3	175	Evolution (29%)	Environmental Sciences (15%)	Ecology (13%)
4	141	Neuroscience (89%)	Medical Sciences (4%)	Biochemistry (2%)
5	117	Biochemistry (43%)	Genetics (20%)	Medical Sciences (14%)
6	92	Microbiology (40%)	Immunology (37%)	Medical Sciences (14%)
7	78	Biochemistry (32%)	Cell Biology (28%)	Medical Sciences (19%)
8	13	Biochemistry (38%)	Medical Sciences (23%)	Neuroscience (23%)

S2 Table. Classification composition of empirically obtained topic communities. Rows represent the eight communities, and columns give the three most common classifications for the topics contained within each community.
