# Supplementary material for: The emergent integrated network structure of scientific research
Source: PLoS One. 2019 Apr 30;14(4):e0216146. doi: 10.1371/journal.pone.0216146 (PMC6490937; doi:10.1371/journal.pone.0216146)
Supplement: S3 Table — Rows represent partitions with between 9 and 16 communities. Columns represent the extent to which the partitions demonstrate modular structure, contain disciplinary communities, and better explain edge weights compared to the classification partition. (PDF) [file pone.0216146.s004.pdf]

# Communities	Modularity (Q)	Disciplinarity	Deviance p-value
9	0.36	0.52	< 0.0001
10	0.35	0.53	< 0.0001
11	0.35	0.53	< 0.0001
12	0.34	0.53	< 0.0001
13	0.34	0.54	< 0.0001
14	0.34	0.55	< 0.0001
15	0.33	0.56	< 0.0001
16	0.33	0.56	< 0.0001

S3 Table. Effect of the number of communities on features of the empirical partition. Rows represent partitions with between 9 and 16 communities. Columns represent the extent to which the partitions demonstrate modular structure, contain disciplinary communities, and better explain edge weights compared to the classification partition.
