# Supplementary material for: The emergent integrated network structure of scientific research
Source: PLoS One. 2019 Apr 30;14(4):e0216146. doi: 10.1371/journal.pone.0216146 (PMC6490937; doi:10.1371/journal.pone.0216146)
Supplement: S4 Table — Rows represent the linear change over time for various null-standardized measures of the temporal network. Columns represent the estimates and statistical significance for different choices of network size. Note: * = p < 0.05, ** = p < 0.01. (PDF) [file pone.0216146.s005.pdf]

Network Measure	N = 950	N = 1000	N = 1050
Strength (*sd* per year)	0.056**	0.056**	0.056**
Unbalanced interdisciplinarity (*sd* per year)	-0.65**	-0.67**	-0.66**
Balanced interdisciplinarity (*sd* per year)	-0.20*	-0.15	-0.13
Small-world propensity (*sd* per year)	0.015*	0.016*	0.014*

S4 Table. Effect of network size on the linear trajectories of the temporal network. Rows represent the linear change over time for various null-standardized measures of the temporal network. Columns represent the estimates and statistical significance for different choices of network size. Note: * = $p < 0.05$, ** = $p < 0.01$.
