# Supplementary material for: The emergent integrated network structure of scientific research
Source: PLoS One. 2019 Apr 30;14(4):e0216146. doi: 10.1371/journal.pone.0216146 (PMC6490937; doi:10.1371/journal.pone.0216146)
Supplement: S5 Table — Rows represent the correlations with PNAS’s impact factor for various measures of the temporal network. Columns represent their values and statistical significance for different choices of network size. Note: * = p < 0.05, ** = p < 0.01. (PDF) [file pone.0216146.s006.pdf]

Correlated Network Measures	N = 950	N = 1000	N = 1050
Small-world propensity - Impact factor (ρ)	0.40	0.39	0.38
Unbalanced interdisciplinarity - Impact factor (ρ)	0.28	0.25	0.17
Balanced interdisciplinarity - Impact factor (ρ)	0.45**	0.41**	0.39**

S5 Table. Effect of network size on the impact factor correlations of the temporal network. Rows represent the correlations with *PNAS*'s impact factor for various measures of the temporal network. Columns represent their values and statistical significance for different choices of network size. Note: * = $p < 0.05$, ** = $p < 0.01$.
