# Supplementary figures and images for: The emergent integrated network structure of scientific research
Source: PLoS One. 2019 Apr 30;14(4):e0216146. doi: 10.1371/journal.pone.0216146 (PMC6490937; doi:10.1371/journal.pone.0216146)

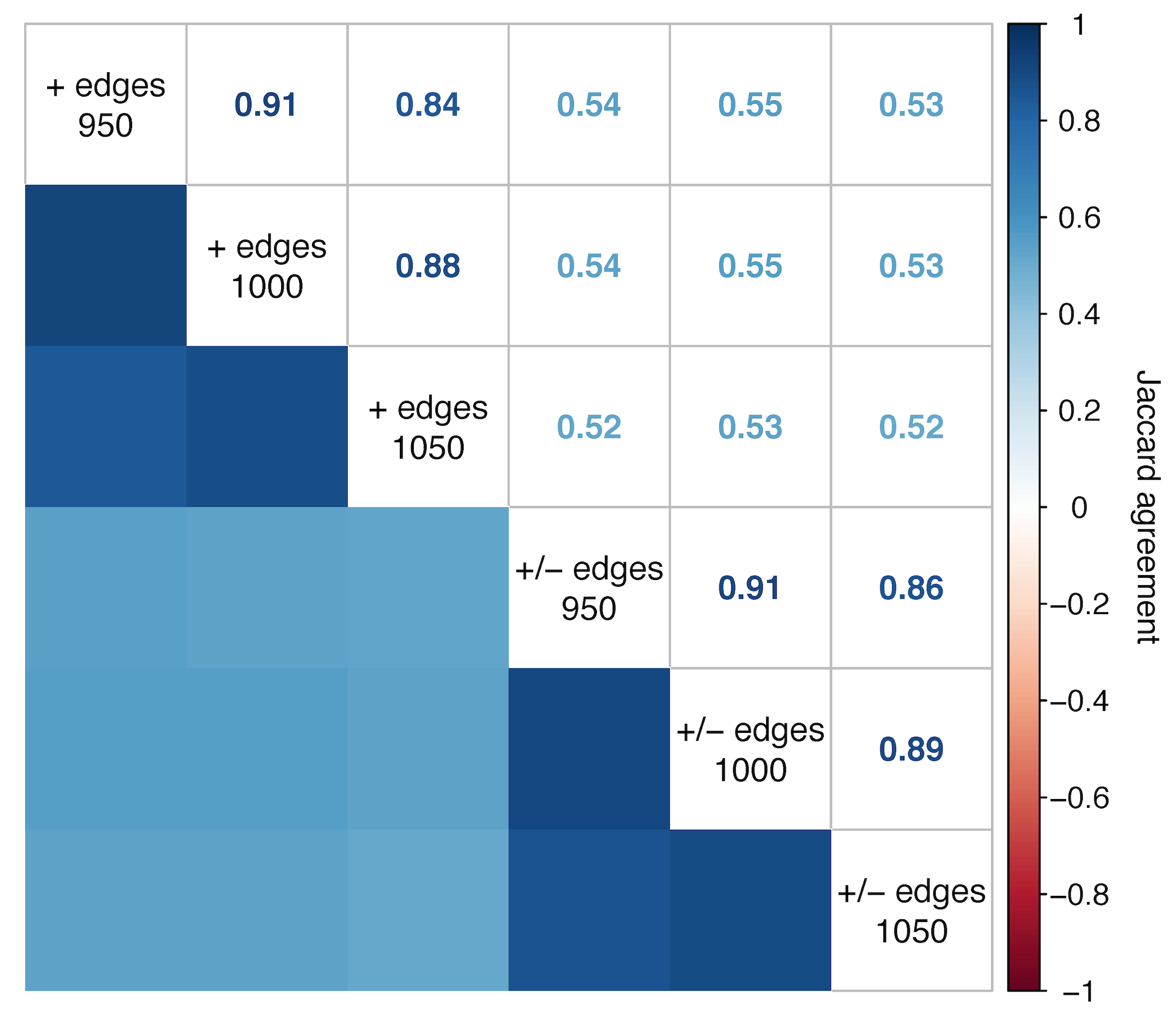

Supplement: S1 Fig — Community structure was consistent across sizes, and was reasonably consistent between positive weighted networks and positive-and-negative weighted networks. (TIFF) [file pone.0216146.s007.tiff]

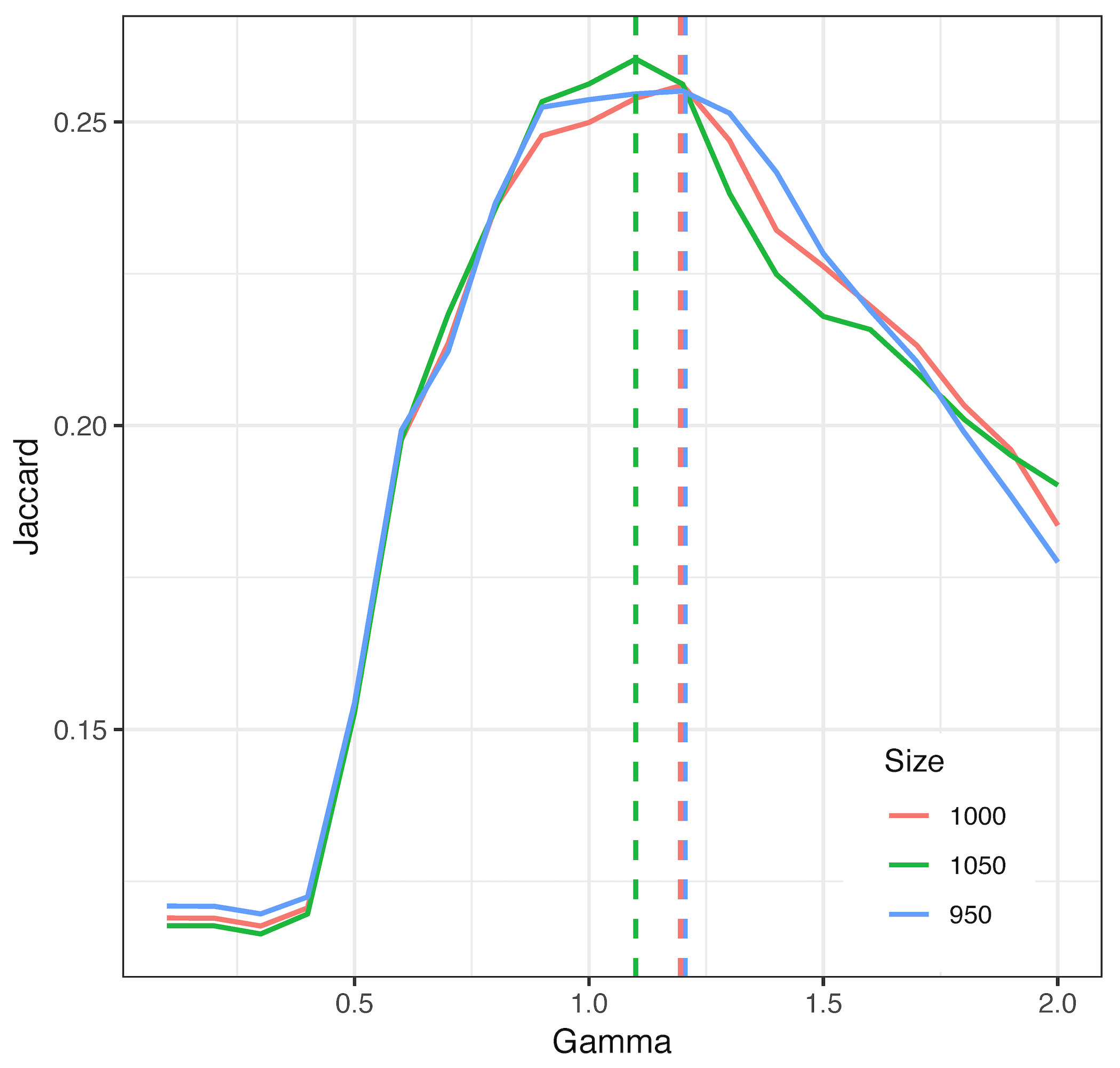

Supplement: S2 Fig — Jaccard similarities are plotted for a range of γ values, demonstrating the procedure for optimizing Jaccard similarity over γ that was used when performing community detection. These values are shown for three different choices of network size. (TIFF) [file pone.0216146.s008.tiff]
